# ORSO (Online Resource for Social Omics): A data-driven social network connecting scientists to genomics datasets

**DOI:** 10.1371/journal.pcbi.1007571

**Published:** 2020-01-24

**Authors:** Christopher A. Lavender, Andrew J. Shapiro, Frank S. Day, David C. Fargo

**Affiliations:** 1 Integrative Bioinformatics, National Institute of Environmental Health Sciences, National Institutes of Health, Research Triangle Park, North Carolina, United States of America; 2 Program Operations Branch, Division of the National Toxicology Program, National Institute of Environmental Health Sciences, National Institutes of Health, Research Triangle Park, North Carolina, United States of America; 3 Office of Scientific Computing, Division of Intramural Research, National Institute of Environmental Health Sciences, National Institutes of Health, Research Triangle Park, North Carolina, United States of America; Johns Hopkins University, UNITED STATES

## Abstract

High-throughput sequencing has become ubiquitous in biomedical sciences. As new technologies emerge and sequencing costs decline, the diversity and volume of available data increases exponentially, and successfully navigating the data becomes more challenging. Though datasets are often hosted by public repositories, scientists must rely on inconsistent annotation to identify and interpret meaningful data. Moreover, the experimental heterogeneity and wide-ranging quality of high-throughput biological data means that even data with desired cell lines, tissue types, or molecular targets may not be readily interpretable or integrated. We have developed ORSO (Online Resource for Social Omics) as an easy-to-use web application to connect life scientists with genomics data. In ORSO, users interact within a data-driven social network, where they can favorite datasets and follow other users. In addition to more than 30,000 datasets hosted from major biomedical consortia, users may contribute their own data to ORSO, facilitating its discovery by other users. Leveraging user interactions, ORSO provides a novel recommendation system to automatically connect users with hosted data. In addition to social interactions, the recommendation system considers primary read coverage information and annotated metadata. Similarities used by the recommendation system are presented by ORSO in a graph display, allowing exploration of dataset associations. The topology of the network graph reflects established biology, with samples from related systems grouped together. We tested the recommendation system using an RNA-seq time course dataset from differentiation of embryonic stem cells to cardiomyocytes. The ORSO recommendation system correctly predicted early data point sources as embryonic stem cells and late data point sources as heart and muscle samples, resulting in recommendation of related datasets. By connecting scientists with relevant data, ORSO provides a critical new service that facilitates wide-ranging research interests.

## Author summary

New sequencing technologies have rapidly transformed biomedical research. Public data repositories now contain millions of datasets, which have the potential to accelerate and bolster research projects. However, the sheer magnitude of available data makes navigation difficult. We created ORSO (Online Resource for Social Omics) to address these challenges. ORSO is a social network where entries are not status updates or tweets, but biological datasets. Users may add their own data to ORSO, joining 30,000 validated datasets that are already hosted, and other users may find these data through intuitive search functions and informative analytics. Users can then favorite datasets relevant to their interests or follow contributing users. ORSO also uses a recommendation system like those used on commercial websites to automatically recommend data to users based on user interactions and dataset similarities. By making data more accessible and by connecting users to relevant data, we anticipate that ORSO will be an important resource for scientists. ORSO may be the first of many applications that use methods originating in social media and ecommerce to enhance and further research projects in the life sciences.

This is a *PLOS Computational Biology* Software paper.

## Introduction

Understanding and contextualizing public data is critical in many research projects. It can focus early hypothesis generation or bolster experimental observations. Advancing technologies and the lowering costs of next generation sequencing (NGS) have led to an exponential increase in available data. Scientists face a volume of public data with a size and complexity that make it difficult to interpret and manage. It is often difficult to find relevant data for even simple queries, such as data originating from a given cell type. Complex queries considering multiple metadata fields, such as ChIP antibody target and cell type, can be even more difficult.

With the rapid proliferation of public genomics data, curation is a persistent and increasingly challenging problem. Outside of large consortial datasets, where consistent protocols and standards are often used, there is little assurance of quality and consistency, even for published data. Many tools have been developed to evaluate data quality, either by calculating sequence quality metrics [1] or by comparisons against validated data [2]. Productive use of data by independent groups may indicate sufficient data quality and be considered as additional validation of a dataset. However, use of data following release is not systematically documented. Citations are indirect, pointing to associated publications and not datasets themselves, and may not describe whether raw or processed data from the original publication are used.

**Fig 1:**
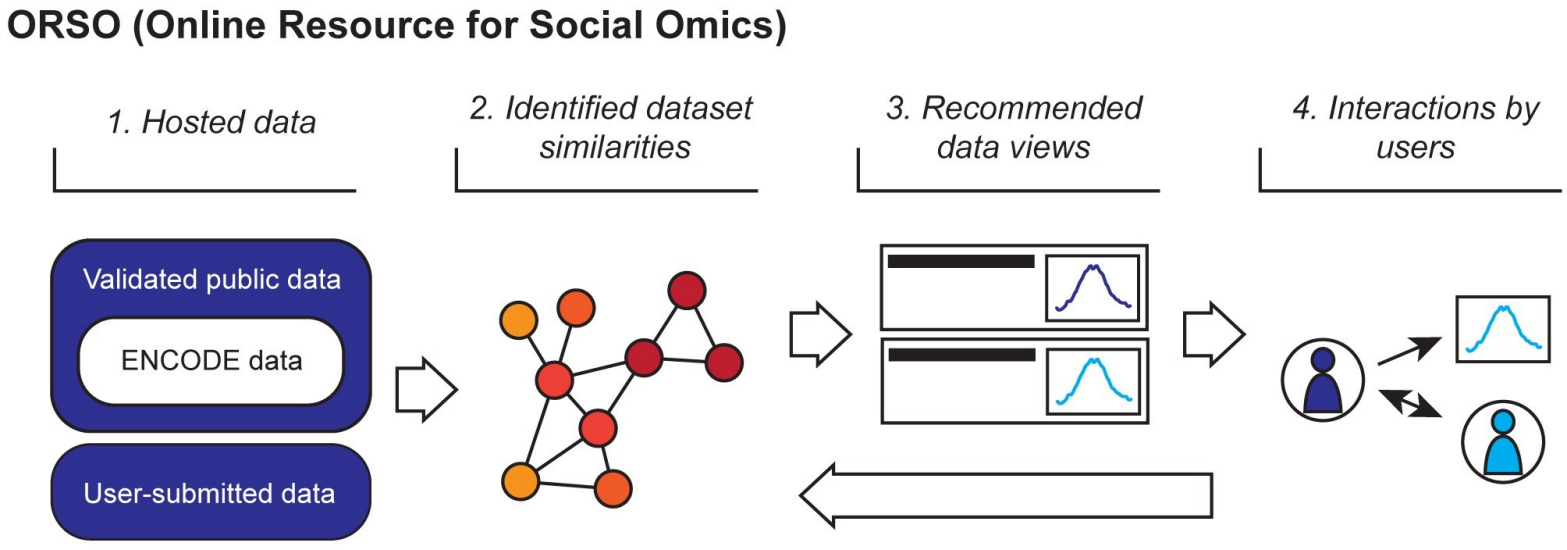
Overview of the ORSO framework. ORSO constructs a data-driven network based on social interactions and identified similarities between datasets. (1) Both validated public data and user-submitted data are hosted by ORSO. (2) Metadata and primary read coverage values are used to construct a data network, where connections represent similarities between datasets. (3) Based on data similarities, individual datasets are recommended to a user based on that user’s interests. (4) User interests are gauged by social interactions with datasets, such as favoriting and following. These interactions are in turn used to connect datasets in the network and impact the data recommended to other users.

To enhance access and utility of public genomics datasets, we present ORSO (Online Resource for Social Omics), a web-based platform for data discovery and evaluation within a social network framework (Fig 1). Using an advanced search engine supporting complex queries over multiple metadata fields, users can find and favorite datasets relevant to their interests. Social interactions, such as favoriting, are used by ORSO to direct a user to new data. First, ORSO presents the number of favorites for each dataset alongside metrics such as gene-by-gene and average coverage values. Data favorited by many users may be amenable to different applications, and these data may be prioritized for interpretation and comparison. Second, social interactions are used in a novel recommendation engine designed to connect users with datasets based on past activity. Inspired by similar recommendation systems used in ecommerce, ORSO presents new datasets to the user based on their interactions with hosted data. Upon favoriting a dataset, similar datasets in the network will be recommended to the user. Dataset similarities are evaluated using primary read coverage values and annotated metadata. Unlike other services [3], ORSO leverages machine learning applications to minimize curation requirements. In addition to direct recommendations, ORSO provides graph-based views of the data network, with datasets as nodes and similarities as edges. These views allow users to explore similarities predicted by ORSO.

ORSO is designed to be an evolving resource. ORSO hosts data from major biomedical consortia, including ENCODE [4], NIH Roadmap [5], modENCODE [6,7], and others [8]. In total, ORSO provides access to over 30,000 datasets from human, mouse, *D*. *melanogaster*, and *C*. *elegans* (summarized in S1 Table). Users can add additional datasets to ORSO. Any dataset added to ORSO is compared in a pair-wise fashion to all other datasets, incorporating it into the ORSO data network, and similar datasets are recommended to the user. Processing and pair-wise analysis have been optimized for rapid integration into ORSO and takes only a few minutes. Users may optionally elect to have their data be public to others, allowing their data to be discovered by other users through search functions or the recommendation engine. As social interactions and other data accumulate through community use, social network-based recommendations will also be more meaningful, leveraging applications traditionally used for business analytics [9].

Research is an inherently social enterprise, with dissemination of results a critical step in any research project. To date, results have largely been disseminated through publication in scientific journals. The acceleration of data generation and technological advances, such as cloud-based computing, are pushing research toward direct dissemination of datasets. Recent changes in publication models acknowledge that data are a key product of research and are in many ways as important as the analysis applied to those data. ORSO anticipates this trend, using a data-centric interconnected network and user-friendly format to empower dataset discovery and contextualization.

## Design and implementation

### Overview of the ORSO web application

ORSO provides a web-based interface to access hosted data and analytics. These views are organized in a tab-based layout (S1 Fig). The “Experiments” and “Users” tabs provide different views to access hosted data and public user profiles. Both tabs provide search functions that allow for complex multi-field queries. The “Explore” tab gives views that allow exploration of all datasets and predicted similarities. These views here include (1) a network view with all datasets shown as nodes in a graph with similarities drawn as connections, (2) a PCA view with dataset read coverage values transformed by principle component analysis, and (3) a dendrogram based on hierarchical clustering of dataset similarities.

ORSO is implemented using Python and Javascript for the backend and frontend, respectively. The web application uses Django framework with data passed to the client via a REST application programming interface (API) [10]. Analytics are performed using Numpy and Scipy [11], with machine learning applications performed using scikit-learn [12] and Keras [13]. Data visualization and graph-based views are implemented using Plot.ly [14] and Sigma [15], respectively. Visualizations made using Plot.ly [14] allow integrated image capture and sharing. All plots use a consistent color scheme for cell types and protein targets. The color scheme can be found at https://github.com/niehs/orso/tree/master/data/colors.

### Adding data to the ORSO framework

ORSO is designed to accommodate NGS data from multiple technologies, including RNA-seq, ChIP-seq, ATAC-seq [16], and others. Users can add new datasets to ORSO using a browser-based form. This form includes fields for required metadata, such as associated assembly and cell type. To create a new dataset, Primary data in the form of read coverage is also required. ORSO does not perform direct upload of primary data. Instead, a user provides an HTTPS-accessible URL to bigWig files containing NGS read coverage [17]. This requirement is consistent with practices common in the field. Popular sequence aligners will generate whole-genome read coverage as optional output files [18], and other tools, such as the popular UCSC Genome Browser, require users to provide HTTPS-accessible bigWig files to display coverage data [19]. Once a dataset is added, ORSO will download the bigWig file and find read coverage values across internally maintained lists of genomic features, including genes and enhancers. Transcripts per million (TPM) values at each feature are saved for downstream analysis. Only feature coverage and metadata fields are saved to the ORSO database; raw coverage information is not retained after processing. After feature counts are found, a dataset is compared against other data from the same experimental technique and organism to find similar datasets.

Genomic features are taken from validated sources in the literature. Feature lists of promoter regions, gene bodies, and mRNA transcripts are taken from RefSeq alignments [20]. Enhancer lists for human and mouse are taken from the VISTA database [21]. Enhancer lists for *D*. *melanogaster* and *C*. *elegans* are taken from validated genome-wide assays [22,23].

ORSO uses a hierarchical relationship to accommodate replicate datasets from the same sample. In short, an “experiment” describing a single biological sample may have one or more “datasets.” This is consistent with the ENCODE project schema [4]. When adding data to ORSO, users have the option of adding multiple datasets to the same experiment. These datasets could be replicates of the same biological sample, or they may be the same replicate aligned to different genome assemblies.

When data are added to ORSO, they may be set as public or private. If a dataset is set to public, that dataset may be discovered by other users, either through the search function or by the recommendation engine. If set to private, a dataset may not be found or accessed by others. However, private datasets are still considered by the recommendation engine, allowing a user to find data similar to an unpublished dataset.

### Dataset comparison methodology

When a dataset is added to ORSO, it is compared against all other datasets from the same organism and experiment type (RNA-seq, ChIP-seq, etc.) to find similar datasets. Independent comparisons are performed using primary read coverage and annotated metadata; datasets may be considered similar by primary data, metadata, or both. Comparisons are knowledge-based, using associations and parent-child relationships from biological ontologies. Different ontologies are used to evaluate different metadata fields, as described below.

For each ontology, we selected important parent classes where children of that class would generally be considered similar. Due to inherent structural differences in each ontology, selections were made manually based on known biology. For instance, parent classes in the BRENDA Tissue and Enzyme Source Ontology were selected to reflect organ system-level organization. Our selections included the parent classes “cardiovascular system” and “neurological system.” Classes such as “head” and “limb” are included to facilitate comparisons across small organisms, such as *D*. *melanogaster* and *C*. *elegans*, where the size of the organisms prevents excision of individual tissues. Parent classes in the Gene Ontology [24] were selected to aid in the identification of transcription factors. A complete list of the key parent classes that were selected can be found in S2 Table.

To facilitate comparisons of histone modifications, we created a custom ontology of epigenetic modifications for use in ORSO. The ontology was adapted from literature [25] and organizes histone modifications based upon genomic locations of enrichments. For example, histone modification H3K4me3 has parent classes “At active promoters” and “At poised promoters”. This custom ontology is available for download at https://github.com/NIEHS/orso/raw/master/ontologies.tar.gz.

### Evaluating metadata similarity

To evaluate similarity by user-annotated metadata, ORSO considers the fields “cell/tissue type” and “target.” The “target” field is a context specific field that depends on experiment type. For instance, in a ChIP-seq experiment, the “target” field would describe the target of the antibody pulldown. For an shRNA knockdown experiment, this would describe the shRNA knockdown target. For some experimental methods, such as RNA-seq or ATAC-seq, the “target” field is not relevant. For two datasets to be considered similar, they must have similar cell types and, if applicable, protein targets.

Metadata comparisons consider key parent classes in the ontology (S2 Table). To evaluate cell type fields, ORSO considers the BRENDA Tissue and Enzyme Source ontology [26]. If two compared cell types share organ system-level parent classes in the ontology, the cell types are considered similar. To evaluate protein target fields, ORSO considers the Gene Ontology [24] and a custom-made histone modification ontology. If key parent classes are shared, the protein targets are considered similar. Because of the nature of this comparison, similarities are recorded and presented in ORSO as categorical values.

An abundance of transcript factors is described in the Gene Ontology. If the two protein target fields are considered transcription factors, ORSO will additionally compare the two targets against the STRING interaction network [27]. Two transcription factors are considered similar only if they show evidence for interaction in STRING.

Metadata evaluation requires user-provided terms to be matched to those in validated ontologies. To mitigate the impact of clerical errors, ORSO uses a “fuzzy” string matching system [28]. In short, the system scores a comparison of characters in two terms. If no direct match is found in an ontology for a given term, ORSO considers ontology terms that exceed a comparison score threshold.

### Evaluating primary data similarity

Similarity based on primary read coverage values are evaluated independently of annotated metadata comparisons. Conceptually, ORSO evaluates similarity of primary data by first predicting metadata classes based on read coverage values and then comparing the predicted metadata of two datasets. Classification models consider the same key ontology parent classes as used in annotated metadata comparisons, meaning that cell type predictions perform organ system-level classification and protein target predictions differentiate between transcription factor and histone modification classes.

Metadata is predicted using a multi-layer perceptron (MLP) neural network [29]. The MLP is trained using read coverage values at genomic features, such as genes. Before applying to the MLP, a filtering step is used to reduce the number of features, removing genes that are constitutively or lowly expressed. Filtering is performed by considering importance in training a random forest classifier to predict cell type or protein target. From an importance-ranked list, the top 1,000 features are taken forward. For each assembly and experiment type, distinct MLPs are trained for each metadata field (e.g. cell type and protein target). To ensure data quality of the initial training set, validated ENCODE data was used to train each MLP model. Training was only performed if at least 100 validated datasets were available. During training, 20% of the datasets were reserved for validation and testing (80%, 10%, and 10% for training, validation, and testing, respectively). Test accuracies are listed in S3 Table.

Variation in MLP test accuracies reflect differences in dataset availability for different experiment types (S1 Table and S3 Table). For instance, only 100 datasets from experiments where CRISPR genome editing was followed by RNA-seq were available for hg19. Training and testing of the MLP associated with these data were performed with only 80 and 10 datasets, respectively. As more datasets are added to ORSO, we expect prediction accuracies to increase.

To evaluate primary data similarity, read coverage values for two datasets are applied to trained MLPs, and cell type and protein target categories are predicted from lists of key ontological classes (S2 Table). Like comparisons of user-annotated metadata, evaluated similarities based on primary data are categorical. If read coverage-predicted cell type and protein target categories are shared, the two datasets are considered similar.

### Recommending datasets through the ORSO data network

After a user adds a dataset to ORSO, that dataset will be compared against all other datasets and incorporated in the ORSO data network. Any similar datasets found will be recommended to the user. Because primary data and metadata are evaluated independently, a dataset may be similar by read coverage, annotated metadata, or both. Users may filter their recommendations such that only datasets with read coverage or metadata similarities are displayed. This may be helpful in situations where annotated metadata may miss important aspects of the experiment design, such as transformation studies where a cell type transition is induced.

### Incorporating user interactions

If a user adds a dataset to ORSO, similar datasets will be recommended to the user. Additional datasets from the ORSO network are also recommended to a user based upon its social interactions. If a user favorites a dataset, datasets similar to the favorite will be recommended to the user. If a user follows another user, that user’s datasets will also be recommended.

Users may only be followed by other users if they set their accounts to public. Users may still use ORSO even if they opt out of using social features. Search functions are available to private users, and private datasets are still considered by the recommendation engine to direct users to similar data.

### Visualization of datasets and data networks

ORSO provides several views for users to browse datasets and perform summary-level evaluations of associated data. For each individual dataset, ORSO provides plots of the average coverage at transcripts, gene bodies, promoters, and enhancers. Read coverage values for each feature are also shown on a scatter plot where they are compared to median values across all datasets of the same assembly and experiment type.

ORSO also provides network-level views to rapidly contextualize datasets. The network is displayed in a graphical map where nodes correspond to datasets and edges correspond to identified similarities. Datasets with the same annotated cell type and protein target are collapsed together, and node size reflects the number of datasets with given cell type and target. Positions of individual nodes are determined using a force-directed layout algorithm [30] such that connected nodes are brought closer together. To provide an additional view, similarity values are applied to a hierarchical clustering procedure, and the resulting dendrogram displays hierarchical relationships between datasets. Additionally, all ENCODE datasets from the same assembly and data type are used to fit a PCA model, and the resulting projection can be used to compare datasets.

## Results

### Recapitulation of known protein interactions and cell type associations

To evaluate the value of ORSO to potential users, we verified that the connections within its network and the content of its displays correctly recapitulated underlying biology. To do this, we used ORSO’s visualization functions to identify meaningful associations in its data network through PCA, network, and dendrogram displays.

**Fig 2:**
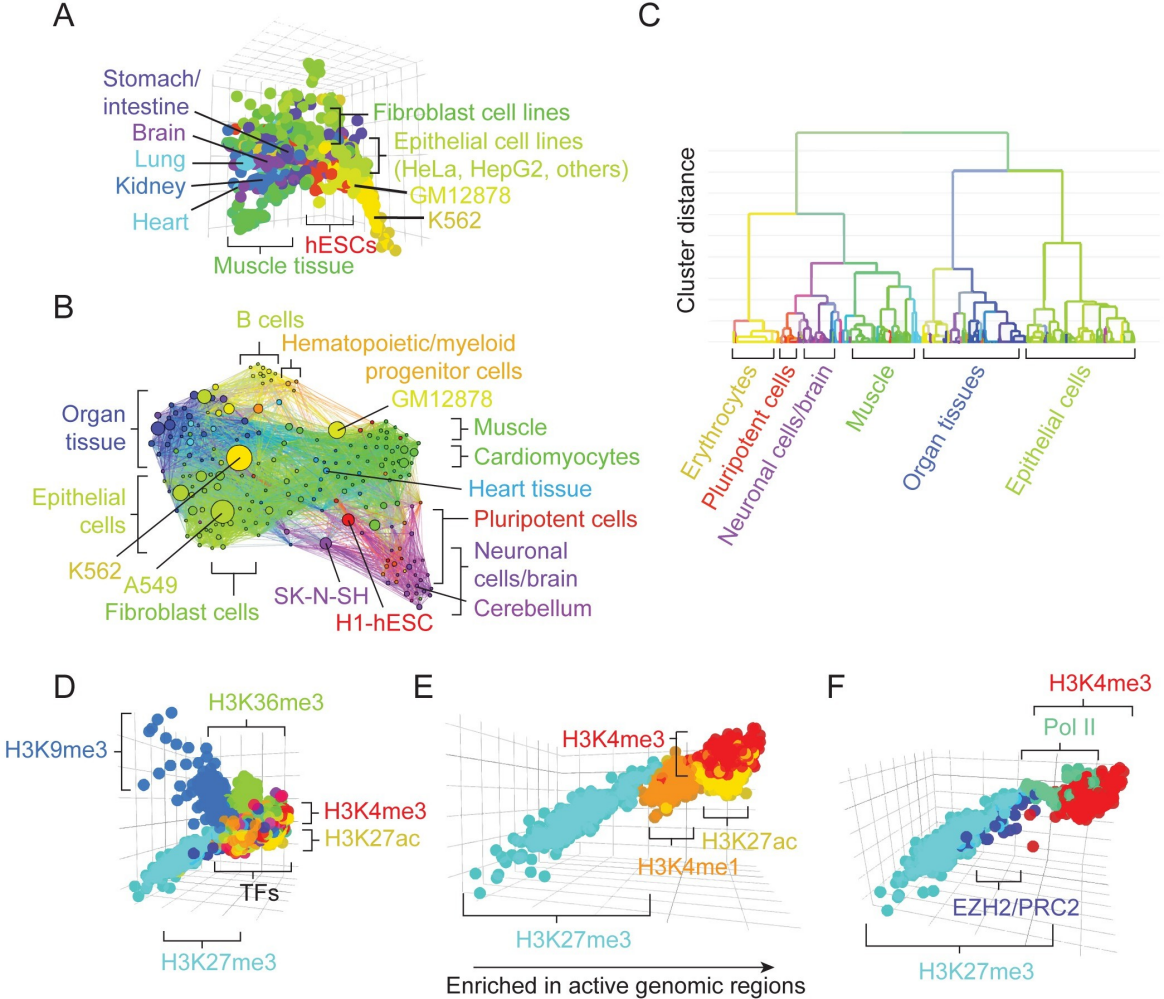
Recapitulation of biological associations in ORSO network and PCA views. All plots were taken from ORSO without modification, except for label overlays. (A) PCA view of human RNA-seq data (hg19 assembly; 1,180 ENCODE datasets). The PCA was constructed considering read coverage values across models of mRNA transcripts. Similar cell types cluster in the same location in PCA space. (B) Network view of human RNA-seq data (805 experiments). Network topologies reflect similarities across cell types. The network layout was generated using a force-directed algorithm that minimizes the distances between connected nodes. (C) Dendrogram view of human RNA-seq data (805 ENCODE experiments). Network similarities were used in hierarchical clustering to create a dendrogram of biologically relevant cell type clusters. (D) PCA view of human ChIP-seq data (hg19 assembly; 4,502 ENCODE datasets). Similar protein targets, including histone modifications, are grouped together in a PCA created using promoter read coverage values. (E) Co-localization of histone modifications associated with active genomic regions in the human ChIP-seq PCA. (F) Co-localization of histone modifications with relevant protein targets in the human ChIP-seq PCA.

We first evaluated human RNA-seq data (an overview of hosted datasets is given in S1 Table). When projected into PCA space, datasets cluster by cell type (Fig 2A). Often, proximity between cell types reflects relevant biology. For instance, datasets from muscle tissue or muscle-derived cells (Fig 2A, green) are proximal to datasets derived from heart (cyan). Related cell types are also co-localized in the network view, indicating that topology of the network map is consistent with biology (Fig 2B). The dendrogram of hierarchical clustering results shows organization of datasets into meaningful clusters (Fig 2C). In both network and dendrogram views, there is evidence of association between organ-derived samples and epithelial cell lines (Fig 2B and 2C, blue and green, respectively). This may reflect the high composition of epithelial cells in samples derived from organs such as kidney and stomach. Co-localization of neuronal and pluripotent cells (Fig 2B and 2C, purple and red, respectively) may indicate similarities in transcriptional programs between these two cell groups.

We then evaluated human ChIP-seq data. The human ChIP-seq PCA space (Fig 2D) is dominated by histone modification datasets, with 44% of datasets from experiments targeting histones (at the time of manuscript preparation, 1995 of 4502 datasets in hg19). Mutually exclusive histone modifications H3K27ac and H3K27me3 segregate to opposite sides of the PCA plot (Fig 2D, yellow and cyan, respectively; detailed in Fig 2E). Other active marks, such as H3K4me3 (in red), aggregate with H3K27ac, while marks associated with repressed gene promoters, such as H3K4me1 (in orange), aggregate with H3K27me3 [25]. Taken together, these marks create an activity axis in PCA space, with active marks on one side and repressive marks on the other. Proximity in PCA space also recapitulates known protein interactions. H3K27me3 datasets co-localize with EZH2 (blue), a member of Polycomb Repressive Complex 2 (PRC2), which deposits this mark (Fig 2F). Associated with active transcription, H3K4me3 associates with RNA polymerase II (Fig 2F, green).

### Evaluating changes during cellular differentiation

**Fig 3:**
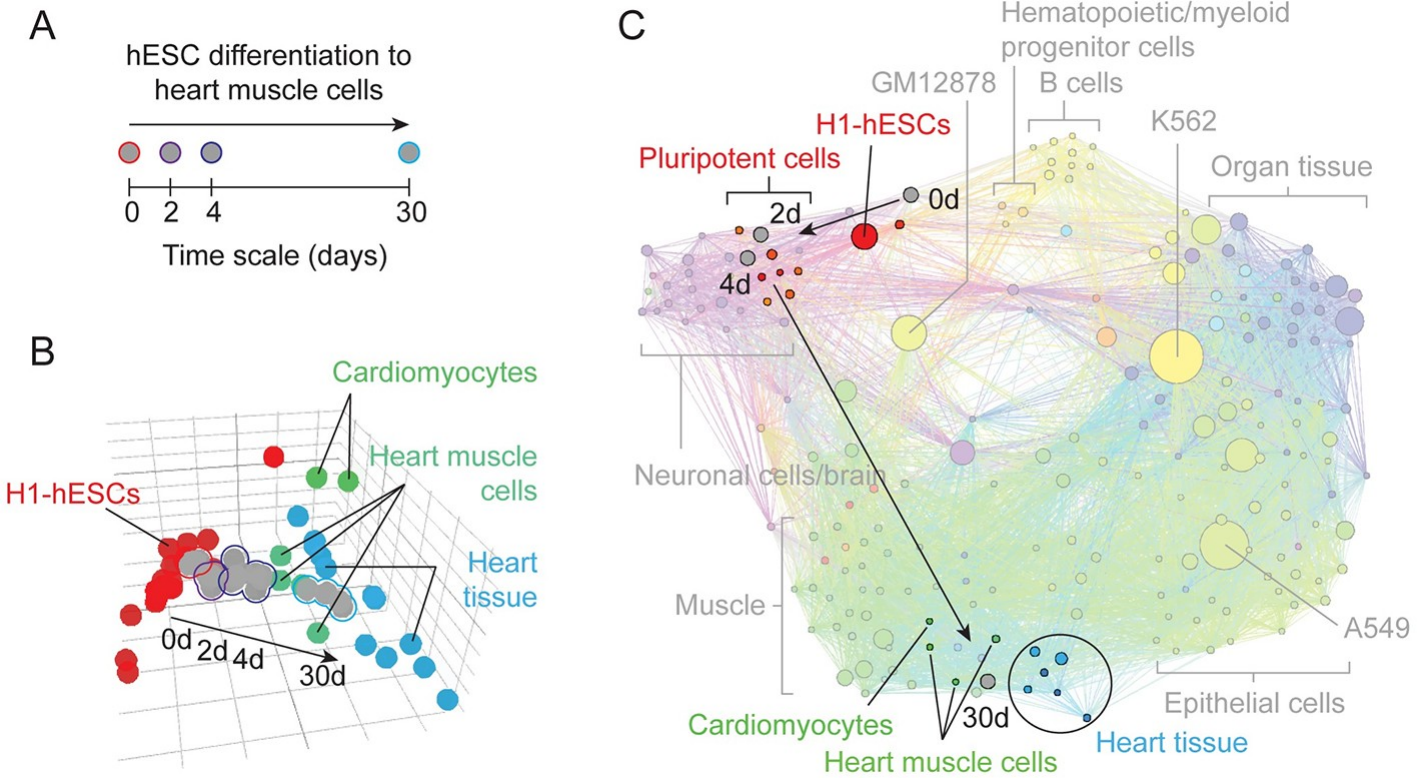
Application of RNA-seq data from a hESC to cardiomyocyte differentiation time course to ORSO. All plots were taken from ORSO without modification, except for label and transparency overlays. (A) Schematic describing the differentiation time course. (B) Differentiation datasets after integration in the human RNA-seq PCA. Early timepoints co-localize with hESCs while later timepoints co-localize with heart muscle samples. (C) Network view after integration of time course data with 805 ENCODE experiments. Localization of timepoints near hESC and heart data points reflect similarities predicted by the ORSO recommendation system.

ORSO allows rapid evaluation and contextualization of user-added datasets in top-down PCA, network, and dendrogram views. To demonstrate the utility of this feature, we added RNA-seq datasets from a time-course experiment [31] measuring differentiation of human embryonic stem cells (hESCs) into cardiomyocytes (Fig 3A; datasets detailed in S4 Table). Each timepoint (0, 2, 4, and 30 days) was added as a distinct dataset. Clear and consistent evidence of differentiation can be seen in the PCA view (Fig 3B). Early timepoints at day 0 (outlined in red) are near other hESC datasets, while later timepoints at day 30 (outlined in cyan) are near datasets associated with the cardiovascular system, including those from heart and cardiomyocytes.

Based on primary coverage information, early timepoints were predicted to be similar to embryonic stem cells while later timepoints were predicted to be similar to samples derived from muscle and heart tissue. These similarities are reflected as connections and placement within the topology of the network view (Fig 3C). Early timepoints are positioned near other embryonic stem cells while the 30-day timepoint is positioned near heart and muscle samples. These network connections are in turn used to recommend datasets to the user (similar datasets are detailed in S5 Table). Upon adding data from early and late differentiation timepoints, ORSO would recommend data to the user from hESCs and cardiovascular cells, respectively.

### Availability and future directions

ORSO is publicly available at https://orso.niehs.nih.gov. Detailed documentation and the complete source code are available at https://github.com/niehs/orso. Included are instructions for Docker-based deployment of an ORSO instance. ORSO is open source software released under the MIT License.

We anticipate ORSO to be refined through continuous development. Given the modular nature of its codebase, additional features may be easily added within the ORSO framework. To greatly expand the number of datasets hosted by ORSO, we hope to develop data validation and natural language processing systems to regularize and incorporate datasets from repositories such as the GEO databank. Current development efforts will introduce gene-based views that allow analysis of enrichment across all datasets and will expand comparison considerations to include fields such as chemical treatment. Additional views could allow the integration of data from multiple experiment types, enabling comprehensive views of the interplay between the epigenome and transcriptome.

Though its social functions are rudimentary compared to commercial social networks, ORSO makes an important step in recognizing the value of measuring the consumption and use of data by scientists. Dataset usage could be valuable in resource allocation and future experiment design. Usage can also be used in a feed-forward strategy to refine ORSO’s recommendation engine. Social interactions may ultimately be combined with data and metadata into a single model that more accurately recapitulates scientist usage patterns.

## Supporting information

S1 Fig The ORSO web interface. The ORSO interface uses a tab-based organization. Each tab brings the user to a collection of views. The “Experiments” tab presents a list of recommended experiments and allows the user to search all experiments hosted by ORSO. Through the “Users” tab, all public user accounts may be searched and accessed. The “Explore” tab gives the user multiple top-down views to explore hosted data. These include a PCA view as well as dendrograms and graph networks constructed from identified similarities between datasets. On all ORSO pages, a “Help” button provides a link to documentation for users and developers.
(TIF)

S1 Table Overview of ENCODE data hosted by ORSO. Datasets are organized by assembly and experiment type. For each assembly and experiment type, the number of datasets and distinct cell types and protein targets are given.
(XLSX)

S2 Table Ontology classes used in metadata comparisons. Listed are key ontology classes whose children are considered similar by ORSO. Parent classes were selected to reflect biological organization of samples. For instance, classes in the BRENDA Tissue and Enzyme Source Ontology were selected to reflect organ system-level organization. For each selected ontology class, the class ID, class name, and ontology are given.
(XLSX)

S3 Table Test accuracies of trained MLP neural networks. MLP models were trained to predict which key parent ontology classes (described in S2 Table) describe a given dataset. Independent models were trained for each combination of experiment type, metadata field, assembly, and genomic feature type. Only validated ENCODE datasets were used to train each model. Datasets were split into training, validation, and test sets using an 80/10/10 split.
(XLSX)

S4 Table Details of an RNA-seq time course of hESC differentiation into cardiomyocytes. The timepoint and cell line are taken from associated GEO repository entries [31]. For each dataset, the ORSO cell type and target metadata fields are given as used in the example vignette.
(XLSX)

S5 Table Dataset recommendations based on time course data. Experiment name and cell type are given for each recommended experiment. Recommended experiments may be similar to multiple experiments from the RNA-seq time course. Multiple datasets are given in a comma-separated list; additional details about these datasets can be found in S4 Table.
(XLSX)
