# Supplementary figures and images for: ORSO (Online Resource for Social Omics): A data-driven social network connecting scientists to genomics datasets
Source: PLoS Comput Biol. 2020 Jan 24;16(1):e1007571. doi: 10.1371/journal.pcbi.1007571 (PMC7001987; doi:10.1371/journal.pcbi.1007571)

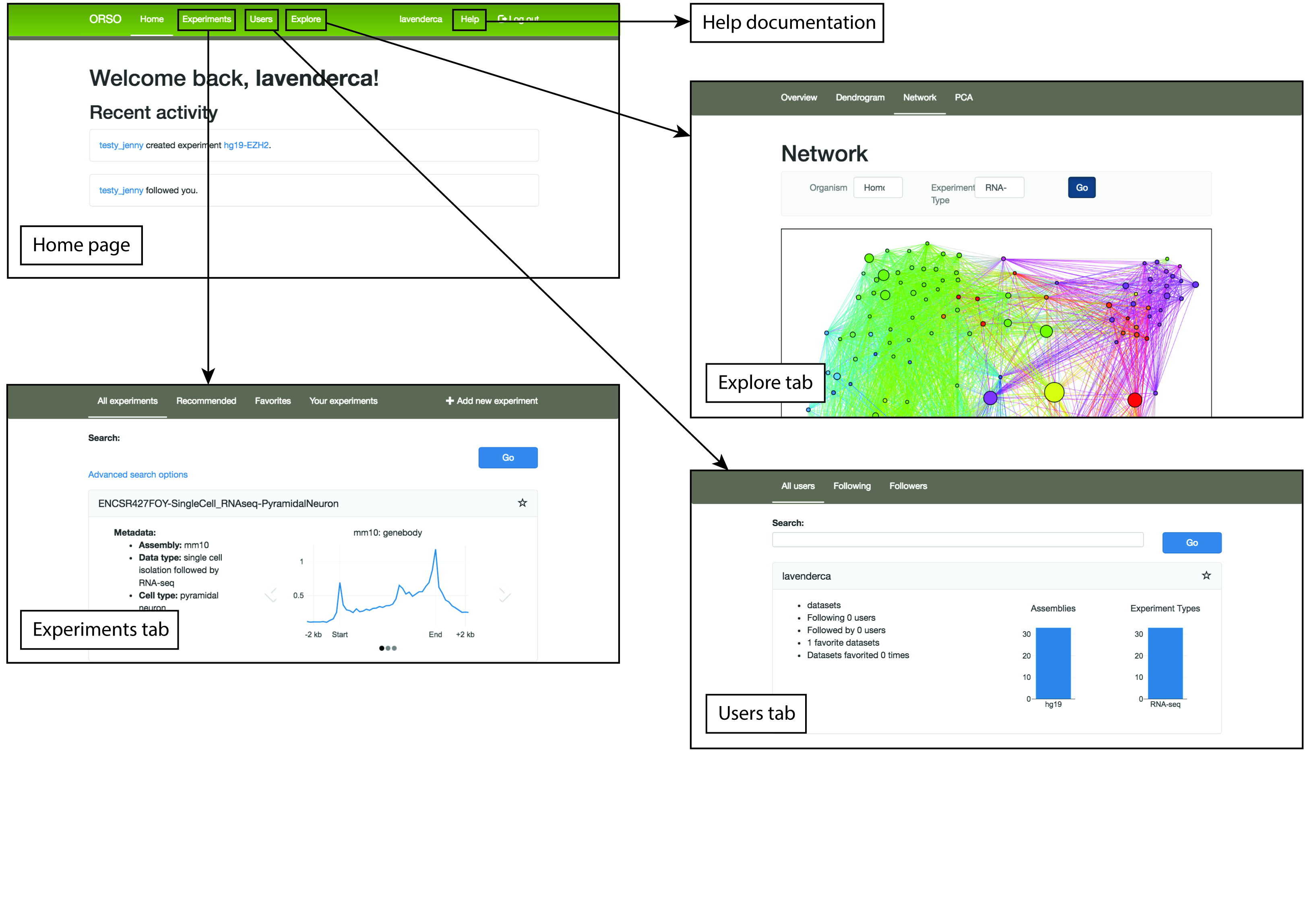

Supplement: S1 Fig — The ORSO interface uses a tab-based organization. Each tab brings the user to a collection of views. The “Experiments” tab presents a list of recommended experiments and allows the user to search all experiments hosted by ORSO. Through the “Users” tab, all public user accounts may be searched and accessed. The “Explore” tab gives the user multiple top-down views to explore hosted data. These include a PCA view as well as dendrograms and graph networks constructed from identified similarities between datasets. On all ORSO pages, a “Help” button provides a link to documentation for users and developers. (TIF) [file pcbi.1007571.s001.tif]
